# CD73 deficiency does not aggravate angiotensin II-induced aortic inflammation in mice

**DOI:** 10.1038/s41598-023-44361-7

**Published:** 2023-10-10

**Authors:** Timo Massold, Fady Ibrahim, Viola Niemann, Bodo Steckel, Katrin Becker, Jürgen Schrader, Johannes Stegbauer, Sebastian Temme, Maria Grandoch, Ulrich Flögel, Pascal Bouvain

**Affiliations:** 1 Experimental Cardiovascular Imaging, Department of Molecular Cardiology, Heinrich Heine University Düsseldorf, Düsseldorf, Germany; 2 Institute for Translational Pharmacology, Heinrich Heine University Düsseldorf, Düsseldorf, Germany; 3 Department of Molecular Cardiology, Heinrich Heine University Düsseldorf, Düsseldorf, Germany; 4 Department of Cardiology, Pulmonology, and Angiology, University Hospital Düsseldorf, Düsseldorf, Germany; 5 Institute for Cardiovascular Sciences, Endothelial Signaling and Metabolism, University Hospital Bonn, Bonn, Germany; 6 Department of Nephrology, University Hospital Düsseldorf, Heinrich-Heine-University Düsseldorf, 40225 Düsseldorf, Germany; 7 Department of Anesthesiology, University Hospital Düsseldorf, Düsseldorf, Germany; 8 CARID, Cardiovascular Research Institute Düsseldorf, Düsseldorf, Germany

**Keywords:** Aortic diseases, Acute inflammation, Preclinical research, Experimental models of disease

## Abstract

Vascular inflammation plays a key role in the development of aortic diseases. A potential novel target for treatment might be CD73, an ecto-5′-nucleotidase that generates anti-inflammatory adenosine in the extracellular space. Here, we investigated whether a lack of CD73 results in enhanced aortic inflammation. To this end, angiotensin II was infused into wildtype and CD73^−/−^ mice over 10 days. Before and after infusion, mice were analyzed using magnetic resonance imaging, ultrasound, flow cytometry, and histology. The impact of age and gender was investigated using female and male mice of three and six months of age, respectively. Angiotensin II infusion led to increased immune cell infiltration in both genotypes’ aortae, but depletion of CD73 had no impact on immune cell recruitment. These findings were not modified by age or sex. No substantial difference in morphological or functional characteristics could be detected between wildtype and CD73^−/−^ mice. Interestingly, the expression of CD73 on neutrophils decreased significantly in wildtype mice during treatment. In summary, we have found no evidence that CD73 deficiency affects the onset of aortic inflammation. However, as CD73 expression decreased during disease induction, an increase in CD73 by pharmaceutical intervention might result in lower vascular inflammation and less vascular disease.

## Introduction

Inflammation of the vessel wall is one of the key factors that initiates and propagates aortic diseases, such as atherosclerosis, abdominal aortic aneurysms (AAA) and dissections (AAD) or deep venous thrombosis. Vascular inflammation is a reactive defense process initiated by chemical, biological or physico-mechanical irritations of the tissue that involves the release of multiple types of extracellular mediators such as cytokines, chemokines, and lipid mediators but also small molecules such as adenosine triphosphate (ATP) or adenosine^1^. These extracellular mediators initiate and modulate the activation and recruitment of immune cells but do also impact structural elements such as the endothelium, fibroblasts or smooth muscle cells. Activated endothelial cells subsequently upregulate the surface expression of molecules such as VCAM1, ICAM1 and E-selectin^2^. In parallel, endothelial cell–cell contacts are loosened, for example, via impact on the VE-cadherin–α-catenin interaction^3^, which enhances the adherence and diapedesis of various immune cells to the vessel wall. On the one hand, vascular inflammation is an important process to eliminate pathogenic microorganisms and to initiate the repair process after chemical or physical tissue damage. On the other hand, uncontrolled or hyperactive vascular inflammation with release of high levels of dangerous effector molecules like reactive oxygen species, or metalloproteases can lead to severe damage of the vessel wall and promote vascular diseases^4,5^.

As mentioned above, initiation and progression of inflammatory reactions are orchestrated by multiple types of extracellular mediators. In recent years, it has been discovered that one important regulatory system of immune reactions are extracellular nucleotides like ATP or adenosine^1^. Whereas extracellular ATP acts as a proinflammatory danger-associated molecular pattern (DAMP) molecule released during processes, such as cell necrosis, extracellular adenosine is mainly considered as an anti-inflammatory molecule^6,7^. Extracellular ATP is rapidly metabolized by cell surface expressed ectoenzymes like CD39 into adenosine monophosphate (AMP)^8^. Additionally, nicotinamide adenine dinucleotide (NAD) is degraded to AMP via CD38 (ADP-ribosylcyclase/cADPR-Hydrolase 1). Both pathways converge at AMP, which can only be converted into adenosine by the 5′-ecto-nucleotidase CD73. Therefore, CD73 is the rate limiting step for the generation of the anti-inflammatory nucleoside adenosine.

Extracellular adenosine can bind to four different adenosine receptors (A1R, A2aR, A2bR and A3R), that belong to the superfamily of G-protein coupled receptors^9^. These receptors are widely expressed on different cell types and exert a large range of functions e.g. modifying the sleep–wake rhythm via A1R on neurons or causing cardioprotective effects on coronary arteries via A3R^10^. Binding of extracellular adenosine to the low-affinity A2bR elicits anti-inflammatory reactions by inhibiting the expression of ICAM1 and E-selectin on endothelial cells and cleaving endothelial cell–cell-interactions^11,12^. Furthermore, A2bR activation down-regulates the IFN-γ-induced MHC class II expression in macrophages^13^ and suppresses oxidase activity in neutrophils^14^. Ligation of the high-affinity A2aR suppresses TNF-alpha and IL12 release by macrophages^15,16^, and inhibits IL12 secretion by both CD4^+^ and CD8^+^ T cells^17–19^.

Multiple studies have investigated the impact of CD73 and CD73-generated adenosine on inflammatory diseases^8^ often by using CD73^−/−^ mice. For example, it has been shown that CD73 plays a crucial role in hypoxia, by increasing vascular leakage and edema formation in the colon, lungs, heart and kidney, promoting organ-dysfunction^20,21^. There is also evidence that CD73 on T cells modulates the healing process after myocardial infarction, as it reduces the release of proinflammatory cytokines like IL6, inhibits fibrosis and reduces infarct size^22–24^. Other vascular diseases seem to be influenced by CD73 as well. In a carotid wire injury model, Zernecke et al.^25^ demonstrated that CD73 protects against neointima formation and vascular inflammation. Furthermore, there is evidence that a lack of CD73 promotes atherosclerosis in ApoE-deficient mice^26^. However, there are also conflicting results. Sutton, Bouïs et al.^27^ could show that the protective effect of CD73 in atherosclerosis changes in older mice, as the observed effect on plaque accumulation reversed in 32–52 weeks old mice.

Although there is a large body of evidence that CD73-derived adenosine has a strong impact on inflammatory diseases, the function of CD73 in early vascular inflammation, which is a key trigger for the development of aortic diseases, has not yet been thoroughly investigated. Therefore, the aim of the present study was to unravel the role of CD73 for the development of inflammatory aortic disease after angiotensin II treatment of wildtype and CD73^−/−^ mice at an early stage when vascular inflammation occurs, preceding the formation of aneurysm. To this end, we analyzed the immune cell infiltration and expression of the ectoenzymes by flow cytometry and also the morphological and functional changes of the aorta using magnetic resonance imaging (MRI), ultrasound and histology in animals with not yet developed AAA. Finally, as sex and age are two important non-manipulable factors for aortic diseases, we further analyzed how the combination of these two factors together with a CD73-deficiency influences early time points of vascular disease.

## Results

### Short-term angiotensin II treatment leads to mild vascular inflammation

**Figure 1:**
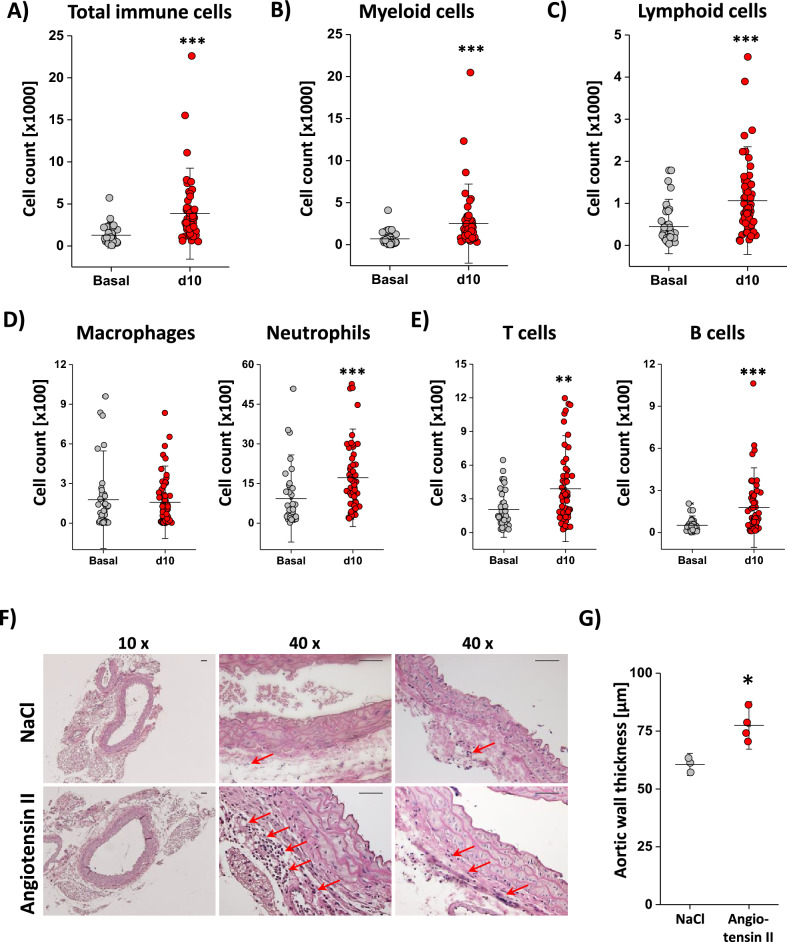
Short-time angiotensin II treatment leads to mild vascular inflammation. (**A**–**E**) Immune cells isolated from the aorta were analyzed by flow cytometry. Dead cells were excluded by DAPI staining and total immune cell numbers (CD45^+^), myeloid (CD45^+^ + CD11b^+^) cells with macrophages (CD45^+^ + CD11b^+^ + F4/80^+^) and neutrophils (CD45^+^ + CD11b^+^ + Ly6G^+^) as well as lymphoid (CD45^+^ + CD11b^−^) cells with T cells (CD45^+^ + CD11b^−^ + CD3^+^) and B cells (CD45^+^ + CD11b^−^ + B220^+^) were determined by flow cytometry. The 10-day treatment with angiotensin II led to a significant increase in total immune cell numbers, which is also visible in myeloid and lymphoid cells as well as neutrophils, T cells and B cells. Only macrophage numbers did not change due to angiotensin II treatment. (**F**) Representative histologic sections of the aorta from mice treated for 10 days by angiotensin II or NaCl. H&E staining revealed substantial immune cell infiltration into the aortic adventitia of mice exposed to angiotensin II as compared with NaCl. Scale bar represents 50 µm in each case (both 10 × and 40 × magnification). (**G**) Aortic wall thickness determined from histologic sections showed a significant increase for angiotensin-II-treated animals compared to NaCl-treated controls. All data sets are mean values ± SD of n = 48–59 (**A**), n = 48–59 (**B**), n = 48–59 (**C**), n = 48–59 (**D**), n = 48–59 (**E**), n = 3–4 (**F**), n = 3–4 (**G**). **p < 0.01 and ***p < 0.001 verified by the Mann–Whitney *U* test (**A**–**E**) or Student’s *t*-test (**G**).

To investigate early aortic disease, we used a mild model of angiotensin II treatment for 10 days via osmotic minipump infusion. Since we were mainly interested in the early time points of aortic disease preceding the formation of substantial anatomical alterations, we excluded all mice which developed an AAA up to day 10 (5–10%). Since we found the variances of this model to be quite high (see Supplemental Table 1), we decided to use a pooling statistical approach to analyze the main effects of angiotensin II treatment, genetic background, age and sex independently of the individual subgroups (see “Methods” for details), which allowed us to keep mouse numbers low in line with the three Rs principle. First, we focused on the impact of angiotensin II treatment and analyzed the early effects on vascular inflammation with regard to number and composition of immune cells within the aortic vessel wall after 10 days of angiotensin II exposure in comparison to saline treatment (Fig. 1). Angiotensin II infusion led to a three-fold increase in total immune cell numbers within the aortic wall (Fig. 1A). Myeloid immune cell counts raised from 700 to 2500 (Fig. 1B) per aorta mainly based on infiltrating neutrophils while changes in macrophage numbers were not detectable (Fig. 1D). Lymphocytes increased from 450 to 1000 cells (Fig. 1C) per aorta with significantly increased total numbers of B and T cells after 10 days of angiotensin II treatment (Fig. 1E).

Next, we subjected aortae from angiotensin II or NaCl-treated mice to histology to verify putative morphological alterations and to investigate the location of infiltrated immune cells within the different aortic layers (Fig. 1F). As can be recognized, this revealed no signs of pathological alterations (e.g. fiber breakdowns) or disintegrity of the various aortic layers. However, measurement of the aortic wall thickness demonstrated a moderate but significant increase in this parameter under angiotensin II exposure (Fig. 1G), which was mainly caused by expansion of the media (Fig. 1F) as also observed in other studies^28^. At the chosen time point (10 days after angiotensin II mini pump implantation) immune cell infiltration was mainly confined to the adventitia indicating neutrophil recruitment from the basal site at this early stage of aortic disease. Interestingly, we found only a trend toward increased neutrophil numbers in blood upon angiotensin II stimulation, which did not reach the level of significance (3.5 ± 1.8 × 10^3^ (basal) vs. 4.9 ± 1.6 × 10^3^ (d10), n = 8–14, n.s.), suggesting that in this model angiotensin II leads to an enhanced recruitment of neutrophils to the adventitia and that this is rather not driven by increased release of immune cells into the circulation.

**Figure 2:**
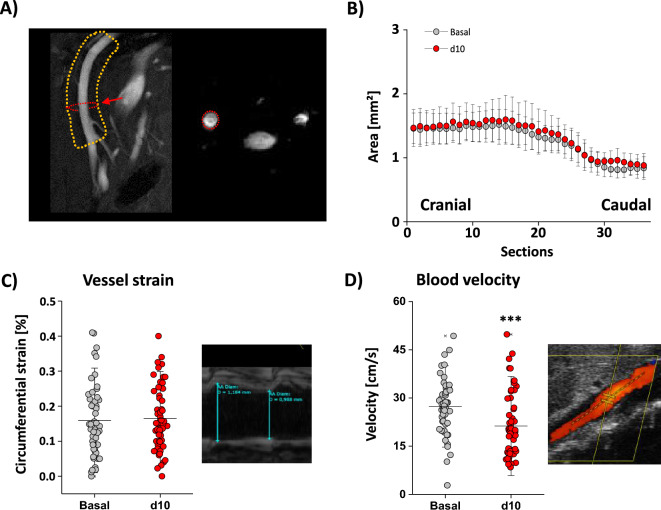
Short-term angiotensin II treatment leads to mild hemodynamic alterations. (**A**) Overview of the abdominal aorta with the area of interest highlighted by an orange rectangle. The cross-sectional area of the aortic blood flow was assessed in axial sections from caudal to cranial by FLASH angio scans. An example of one section is highlighted by a red circle and the corresponding transverse image is given on the right. (**B**) Quantification of the sectional areas of the aorta to determine the vessel lumen. We could not detect any differences between basal measurements (grey) and after 10 days of treatment with angiotensin II (red) in the vessel lumen. (**C**) Strain of the vessel was determined by ultrasound examination. While quantification is given on the left with no alterations between basal (grey) and d10 (red) an exemplary measurement is shown on the right. (**D**) The blood flow velocity was also determined by ultrasound examination. Here, quantification on the left revealed a significant drop in blood flow velocity due to the treatment of angiotensin II for 10 days. An example of the measurements is shown on the right. All data sets are mean values ± SD n = 37–41 (**B**), n = 58 (**C**) and n = 58 (**D**). ***p < 0.001 verified by the Mann–Whitney *U* test.

Since we observed the development of significant aortic inflammation during the short observation period, we wondered if this is also associated with alterations in aortic lumen, vessel strain or the blood flow velocity after angiotensin II treatment. The aortic lumen was determined via MRI in an area 3 mm below and up to 6 mm above the inferior renal vessel which is prone to develop AAA or AAD in mice. In Fig. 2A left, an anatomical overview is provided, where the area of interest is highlighted by an orange rectangle and an example of an axial scan indicated by a red circle is shown on the right. With this imaging technique, we found no alterations in arterial lumen after a 10-day treatment with angiotensin II in comparison to the basal values as indicated by the sectional analysis of the vessel lumen from cranial to caudal (Fig. 2B). To have a more detailed look at vessel function, the aortic wall strain as well as the mean velocity of the blood were determined by ultrasound. Both parameters were evaluated below the coeliac and above the cranial mesenteric artery. Strain analysis revealed no changes in elasticity of the aortic wall (Fig. 2C). However, blood flow velocity was slightly reduced on day 10 of angiotensin II treatment (Fig. 2D). In total, we demonstrate that the used model is indeed suitable to study processes involved in early aortic disease, in that we observed a significant increase in immune cell infiltration into the aortic wall, mainly due to neutrophils, and a small decrease in blood flow velocity but without any changes in vessel lumen or aortic strain.

### Angiotensin II treatment leads to downregulation of CD73 on vessel-infiltrating immune cells

**Figure 3:**
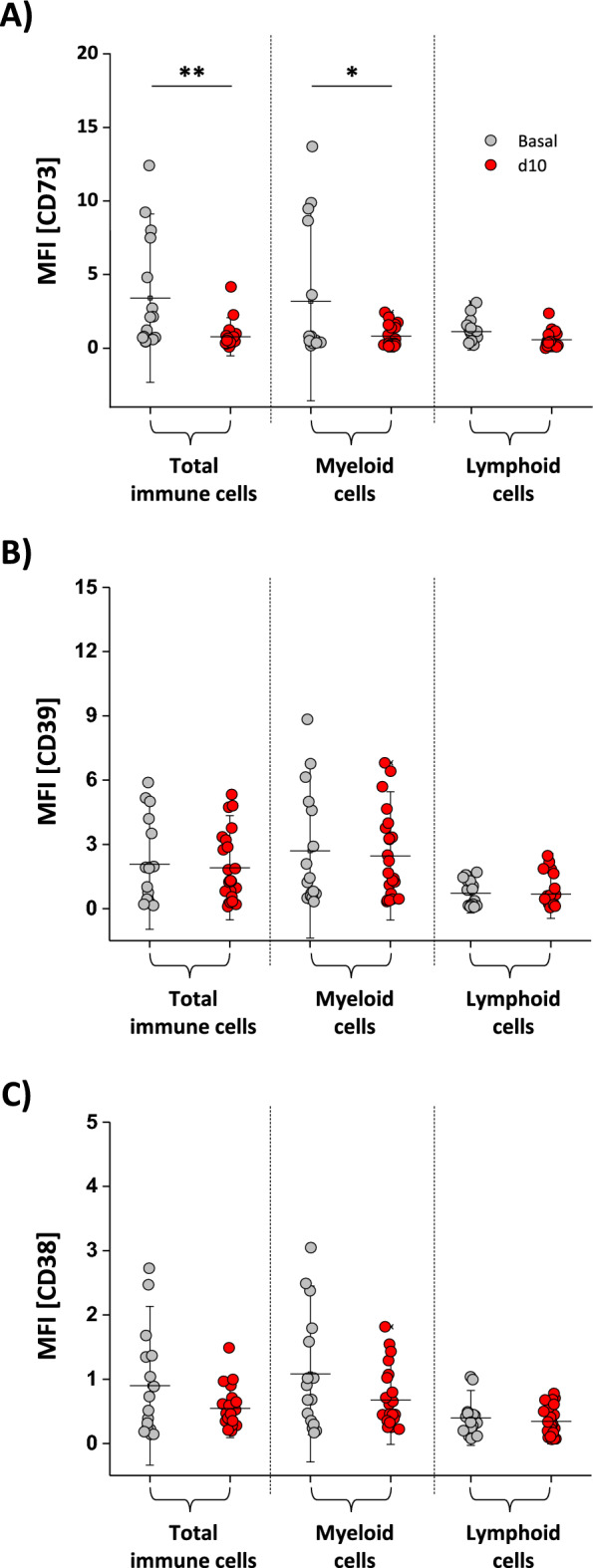
Inflammation-dependent downregulation of CD73. The surface expression of CD73 (**A**), CD39 (**B**) and CD38 (**C**) was determined on total immune cells (top) as well as on myeloid (middle) and lymphoid (bottom) cells isolated from the aorta from CD73 WT mice under basal conditions (grey) and 10 days after angiotensin II treatment (red) via flow cytometry. We detected a significant decrease of CD73 on total immune cells and myeloid cells after 10 days of angiotensin II treatment with no alterations of the other two ectoenzymes (CD38, CD39). All data sets are mean values ± SD of n = 16–23 (**A**), n = 16–23 (**B**) and n = 16–23 (**C**). *p < 0.05 and **p < 0.01 verified by the Mann–Whitney *U* test.

As CD73, the key enzyme for degradation of AMP to adenosine, has been described to be involved in several cardiovascular diseases^22–24^, we subsequently investigated whether CD73 is also linked to early vascular inflammation in the present model. To this end, only data for wildtype mice were analyzed with regard to surface expression of CD73 on total immune cells as well as on myeloid and lymphoid cells. As shown in Fig. 3A, there was a significant decrease in CD73 on total immune cells by a factor of 4. Similar observations were made for myeloid cells (reduction of surface CD73 by a factor of 4) while lymphoid cells exhibited no reduction in surface expression of CD73 and had less CD73 than the myeloid cells in general (Fig. 3A). In a more detailed analysis, we found a significant downregulation of CD73 on isolated neutrophils from the aorta after angiotensin II treatment while T and B cells exhibited similar expression patterns independent of angiotensin II treatment (see Supplemental Fig. S1). Since neutrophils were characterized by substantial higher expression levels of CD73 compared to other immune cells (T cells, B cells; Supplemental Fig. S1) and are the predominant immune cell type infiltrating the aortic wall (Fig. 1D + E), immune cell dependent adenosine metabolism seems to be primarily dependent on this cell type. Because of the low cell numbers of macrophages (often less than 100 cells per aorta), reliable assessment of surface expression of ectoenzymes was not possible for this cell type.

As mentioned above, CD73 is the key enzyme to degrade AMP to adenosine and is considered the bottle neck enzyme for AMP breakdown. Since we found CD73 downregulated after the 10-day treatment with angiotensin II, we next verified the expression of AMP-providing enzymes. The two main ectoenzymes which are necessary to produce AMP are CD39 (conversion of ATP over ADP to AMP) and CD38 which degrades NAD over CD203a to AMP. Thus, we determined the surface expression of CD38 and CD39 on isolated immune cells from the aorta for control animals and angiotensin II treated ones via flow cytometry. However, neither of the two enzymes exhibited a change in surface expression on total immune cells or the myeloid/lymphoid cell line after angiotensin II treatment (Fig. 3B + C). Similar results were observed for T and B cells, but interestingly we found also a significant downregulation of CD38 and CD39 on neutrophils (see Supplemental Fig. S1). To answer the question if angiotensin II by itself can change the ectoenzyme expression of CD73, we incubated murine blood leukocytes with different angiotensin II concentrations for one hour and determined the surface expression of CD73 via flow cytometry in comparison to cells incubated only in medium. As shown in Supplemental Fig. S2, we could not detect any angiotensin II-dependent change in surface expression of CD73 on blood immune cells excluding a direct effect of angiotensin II on CD73 surface expression.

In summary, we found no alterations in AMP-producing enzymes (CD38, CD39) upon angiotensin II treatment but a significant decrease in AMP-degrading ones (CD73), especially on neutrophils.

### CD73^−/−^ does not lead to changes in vessel function or immune cell infiltration

**Figure 4:**
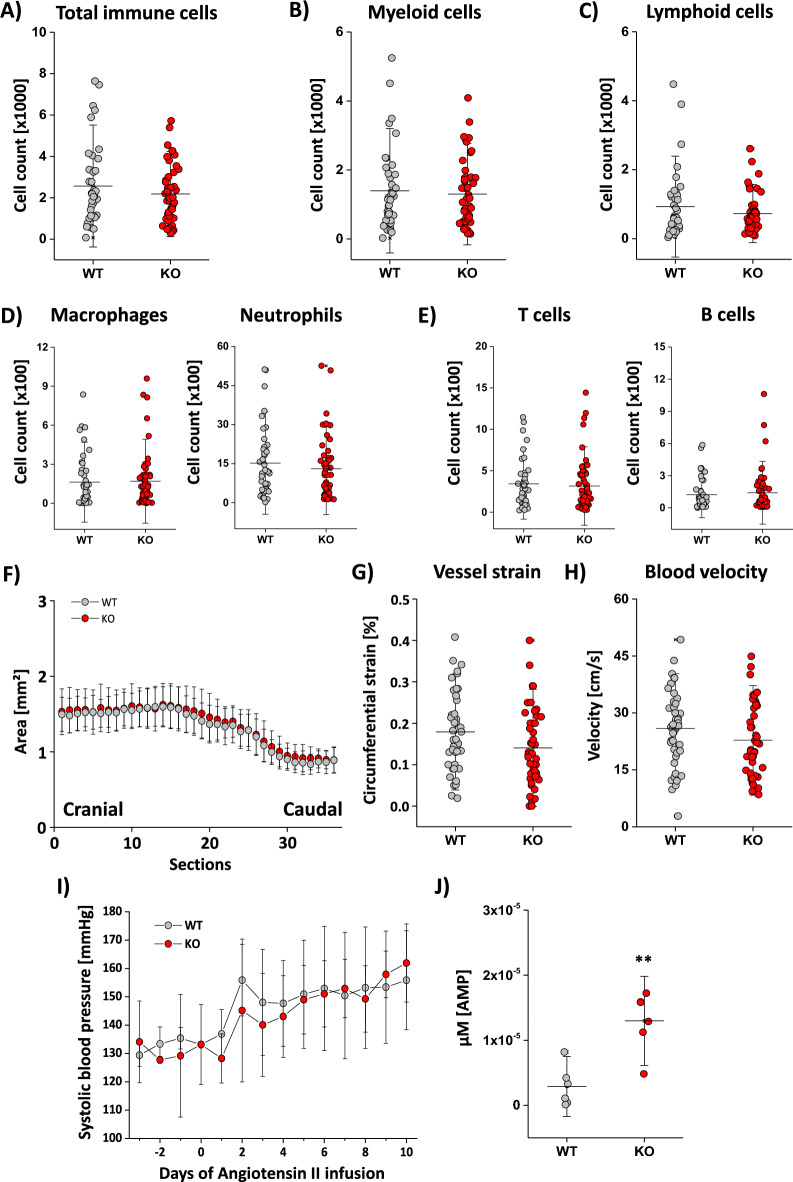
CD73 deficiency does not aggravate angiotensin II induced vascular inflammation in mice. (**A**–**E**) Investigating the immune cell distribution numbers within the vessel wall, we could not detect any difference in total immune cells (**A**), myeloid (**B**) or lymphoid (**C**) cells between CD73 wildtype (grey) and CD73^−/−^ animals (red) after 10 days of angiotensin II treatment. The same was true for macrophages and neutrophils (**D**) or T and B cells (**E**) when comparing CD73 wildtype (grey) and CD73^−/−^ animals (red) after angiotensin II exposure. (**F**) Vessel area was determined by axial FLASH scans via MRI. Quantification revealed no differences between CD73 wildtype (grey) and CD73^−/−^ (red) mice. (**G**) Vessel strain, determined by ultrasound examination, showed no differences between CD73 wildtype (grey) and CD73^−/−^ (red) animals. (**H**) Blood flow velocity, also determined by ultrasound examination, again showed no differences between the CD73 wildtype (grey) and CD73^−/−^ (red) animals. (**I**) Temporal development of systolic blood pressure in CD73 wildtype (grey) and CD73^−/−^ (red) mice after minipump implantation with angiotensin II (at day 0). (**J**) Isolated immune cells from the aorta were incubated with ATP and the degradation products (e.g. AMP) were determined via UPLC measurements. All data sets are mean values ± SD of n = 39–47 (**A**), n = 39–47 (**B**), n = 39–47 (**C**), n = 42–54 (**D**), n = 42–54 (**E**), n = 36–42 (**F**), n = 44–48 (**G**), n = 44–48 (**H**) and n = 6 (**I**).

As demonstrated above, the short-term treatment with angiotensin II led to early vascular inflammation characterized by increased immune cell recruitment into the vessel wall and was accompanied by decreased CD73 expression, especially on the neutrophil cell surface. To answer the question if a lack of CD73 will have any functional impact on the early vascular inflammation, we analyzed the data with regard to the genetic background and compared wildtype with CD73^−/−^ mice after treatment with angiotensin II for 10 days. To our surprise, we could not detect any changes between these groups in total immune cell numbers (Fig. 4A) or in myeloid (Fig. 4B) and lymphoid cells (Fig. 4C) which infiltrated the vessel wall due to the angiotensin II treatment. Similar results were found when comparing macrophages, neutrophils and T and B cells from wildtype and CD73^−/−^ mice (Fig. 4D + E). In line with this, we also could not detect any differences in the vessel lumen (Fig. 4F), the wall strain (Fig. 4G) or blood flow velocity (Fig. 4H) in CD73^−/−^ mice compared to wildtype animals. To exclude any genetically driven effects, we investigated CD73 WT and KO also under basal conditions with no changes for immune cells within the aorta or any differences in blood flow and strain (see Supplementary Fig. 3). As expected, systolic blood pressure rose over the 10-day treatment with angiotensin II, independent of the genetic background (Fig. 4I). To investigate if CD73 is the only enzyme that degrades AMP, immune cells from the aortae of CD73-deficient mice were isolated and incubated for 30 min with ATP. Afterwards, degradation products of ATP (e.g. AMP) were determined within the supernatant via UPLC measurements. Quantification of AMP level in the supernatant revealed significantly more AMP within CD73^−/−^ in comparison to wildtype animals (Fig. 4J).

### Age and gender as main non-manipulable factors for development of aortic disease

**Figure 5:**
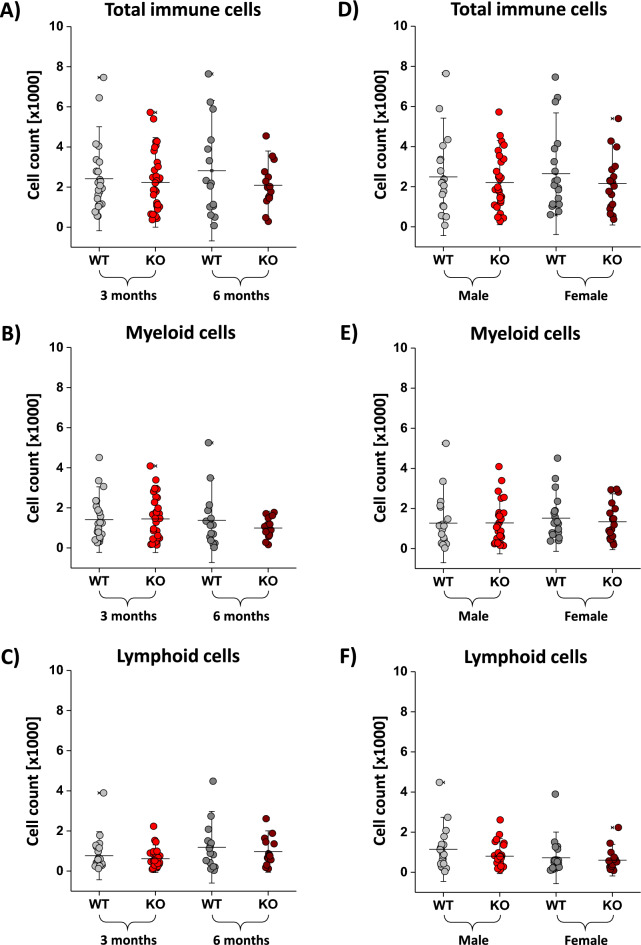
Risk factors age and gender did not influence immune cell numbers in dependency of CD73^−/−^. (**A**–**C**) Immune cells were isolated from the aorta from 3 (bright) and 6 (dark) months old wildtype (grey) and CD73^−/−^ (red) mice. We did not see any differences between total immune cells (**A**) or the myeloid (**B**) or lymphoid (**C**) cells. (**D**–**F**) Immune cells were isolated from the aorta from male (bright) and female (dark) wildtype (grey) and CD73^−/−^ (red) mice. We did not see any differences between total immune cells (**D**) or the myeloid (**E**) or lymphoid (**F**) cells. All data sets are mean values ± SD of n = 15–32 (**A**), n = 15–32 (**B**), n = 15–32 (**C**), n = 15–32 (**D**), n = 15–32 (**E**) and n = 15–32 (**F**).

Formation of AAA is marked by different manipulable and non-manipulable risk factors. The two major non-manipulable risk factors are gender and age. In general, male and elderly persons are more prone to develop AAA. To investigate whether early vascular inflammation is affected by age and gender, we compared the two main factors age (3 and 6 months) as well as sex (male and female) for wildtype and CD73^−/−^ mice after treatment with angiotensin II for 10 days regarding their vessel lumen, wall strain and blood flow velocity as well as immune cell composition in the aortic wall. However, we could not detect any alterations in the immune cell composition in aged CD73^−/−^ and wildtype mice (Fig. 5A–C). This was also mirrored in a more detailed analysis of the different immune cell populations (macrophages, neutrophils, T and B cells) shown in Supplemental Fig. S4. While neutrophils were the main contributors to aortic inflammation, cell numbers of macrophages and T and B cells was 10 times lower. Similar results were seen when male and female mice were compared in a wildtype and CD73^−/−^ background (Fig. 5D–F). Neither of the changes in the total number of immune cells nor in the myeloid or lymphoid cell numbers were visible in the more detailed analysis of macrophages, neutrophils, T and B cells (see Supplemental Fig. S5). In summary, age as well as gender do not seem to play any role in the early vascular inflammation in a CD73-driven background.

**Figure 6:**
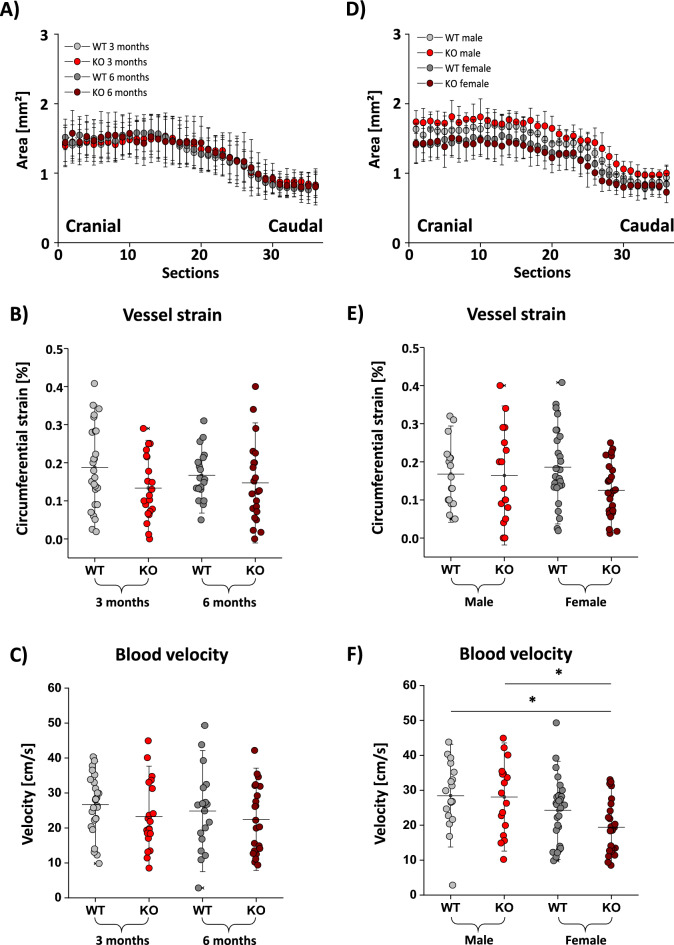
Risk factors age and gender do not influence vascular physiology in dependency of CD73^−/−^. (**A**) Vessel lumen was determined by axial FLASH scans via MRI. Quantification revealed no differences between 3 (bright) and 6 (dark) months old CD73 wildtype (grey) and CD73^−/−^ (red) mice. (**B**) Vessel strain was determined by ultrasound examination with no significant differences between 3 (bright) and 6 (dark) months old CD73 wildtype (grey) and CD73^−/−^ (red) animals. (**C**) Blood flow velocity was determined by ultrasound examination with no significant differences between 3 (bright) and 6 (dark) months old CD73 wildtype (grey) and CD73^−/−^ (red) mice. (**D**) Vessel lumen was determined by axial FLASH scans via MRI. Quantification revealed no differences between male (bright) and female (dark) months old CD73 wildtype (grey) and CD73^−/−^ (red) mice. (**E**) Vessel strain was determined by ultrasound examination with no significant differences between male (bright) and female (dark) CD73 wildtype (grey) and CD73^−/−^ (red) animals. (**F**) Blood flow velocity was determined by ultrasound examination with no significant differences between male (bright) and female (dark) months old CD73 wildtype (grey) and CD73^−/−^ (red) mice. All data sets are mean values ± SD of n = 8–17 (**A**), n = 10–14 (**B**), n = 19–27 (**C**), n = 19–27 (**D**), n = 19–27 (**E**) and n = 19–27 (**F**). *p < 0.05 verified by the Kruskal–Wallis test.

Furthermore, we checked defined vascular parameters via MRI and ultrasound scans e.g. aortic lumen, strain and blood flow velocity. The factor age showed no CD73-related influence on the vessel lumen (Fig. 6A) nor the strain (Fig. 6B) or blood flow velocity (Fig. 6C). However, we detected a trend to a decrease in vessel lumen in both female CD73 WT and CD73^−/−^ mice (Fig. 6D), which proved significant when the entire area under the curve was analyzed (see Supplemental Fig. S6). A trend was also seen in the wall strain analysis (Fig. 6E), while blood flow velocity significantly decreased in female CD73^−/−^ mice in comparison to male CD73^−/−^ and wildtype mice (Fig. 6F).

## Discussion

Within this study we could demonstrate that short-term angiotensin II treatment in mice that are not deficient in ApoE or treated with a high-fat diet results in mild aortic inflammation without inducing major structural alterations in the aorta, making this model suitable for studying early processes causing aortic disease and, in particular, the first steps of AAA/AAD formation. Furthermore, we demonstrate that lack of CD73 does not influence vessel anatomy or immune cell content in the aortic wall in general and after angiotensin II treatment. Two main risk factors for the development of aortic disease (age and gender) were also not influenced by the lack of CD73. Even compensatory effects, like changes in ectoenzyme expression of CD73-linked enzymes (e.g. CD38 or CD39), were not altered during inflammation. However, we found a downregulation of CD73 on immune cells, especially neutrophils, infiltrating the vessel wall, indicating an angiotensin II dependent switch to a pro-inflammatory state.

Angiotensin II is a vasoactive hormone and part of the Renin–Angiotensin–Aldosterone system (RAAS) crucially involved in regulation of liquid and electrolyte balance and, thus, targeted in modern antihypertension therapy. Acting via the AT1 and AT2 receptors, angiotensin II mediates a variety of processes in very different tissues, primarily serving to increase blood pressure as well as blood sodium levels^29^. However, angiotensin II can also modulate immune cell functions, e.g. trigger the proliferation of splenic lymphocytes via the AT1 receptor and mobilize macrophages of the spleen to promote the development of aortic aneurysm^30,31^. In mice, angiotensin II treatment—first described in 2000^32^—has become the most popular model for AAA/AAD, used in a wide range of treatment regimens with up to 12 weeks of angiotensin II exposure and with different genetic backgrounds, mainly based on ApoE deficiency^33^. Here, angiotensin II treatment results in severe histopathological impairments such as leucocyte infiltration, degeneration of the media, atherosclerosis, intramural thrombus, and wall dissection^34,35^.

In the present study, we used a short-term angiotensin II treatment period of 10 days only and a non-ApoE background to focus on leucocyte infiltration during the early onset of aortic disease where major macroscopic changes of the aortic vessel wall were not yet present. The comprehensive characterization of this model by various methods (MRI, ultrasound, flow cytometry, histology) demonstrated the suitability of our approach to identify initial processes contributing to the subsequent formation of severe aortic diseases. We showed that short term angiotensin II treatment resulted in significant vascular inflammation and an increased infiltration of immune cells into the aortic wall without having altered the vessel anatomy and stiffness. The results after treatment with angiotensin II showed a threefold increase in the total number of immune cells within the aortic wall with the most pronounced alterations for infiltrating neutrophils and almost no changes in the macrophage cell count. Of note, neutrophils are considered to be important effector cells in the innate arm of the immune system^36^ and our observations are in line with a study by Mellak et al., where in mice that did not develop AAA after angiotensin II exposure, monocyte levels did not increase and remained stable over time^31^. In contrast, neutrophils were recruited into the vessel wall at an early time point and displayed high levels of cytokine release, NET formation and granule protein secretion^37,38^. Despite the significant changes in immune cell distribution, we found no association with anatomical or hemodynamic alterations. Interestingly, angiotensin II treatment resulted in a slightly lower blood flow velocity at the end of the observation period. In this context, Gasparo et al. discussed an activation of AT_2_ receptors, especially found in the kidney, enhancing the synthesis of vasodilative NO, which may be the reason for this decrease in blood flow velocity^39^.

The 5′-ecto-nucleotidase CD73 is considered to be the bottle neck for the generation of the anti-inflammatory nucleoside adenosine, since it is the main enzyme to break down extracellular AMP. Considering the known anti-inflammatory properties of adenosine, one could expect that the CD73^−/−^ mice would display strong levels of vascular inflammation. This notion is supported by various studies showing that lack of CD73 accompanied by decreased adenosine levels is linked to chronic inflammatory diseases^40^ and increased infiltration of immune cells, which is a major risk factor for the development of AAA^41,42^. Eckle et al. showed that genetically targeted mice with defects in CD73 or CD39 exhibited an increased susceptibility to acute lung injury^43^. Zernecke et al. demonstrated that CD73 is crucially involved in the finely tuned constitutive regulation of balancing proinflammatory and anti-inflammatory mechanisms in the microvasculature, therefore offering a protective effect against vascular inflammation and neointima formation^25^. In another study from Hart et al., it was shown that pharmacological inhibition or genetic deletion of CD73 due to decreased adenosine generation significantly increased infiltration of neutrophils into the intestinal tissue following intestinal ischemia–reperfusion^44^. To our surprise, we could not detect any detrimental effects in this early phase of aortic disease when CD73 was lacking, despite being linked to a variety of different disease patterns, as mentioned above.

Interestingly, we found a decrease of CD73 expression on total immune cells infiltrated into the aortic wall after 10 days of angiotensin II exposure in wildtype mice. In particular, there was a significant downregulation of CD73 on neutrophils, while T and B cells exhibited similar expression patterns after angiotensin II treatment as compared to baseline conditions. Neutrophils were the predominant immune cells found in the aortic wall and also displayed the highest expression levels of CD73 in comparison to all other immune cell types. As a consequence, adenosine levels within the aortic wall are expected to be linked to the number of neutrophils that infiltrated into the inflammatory microenvironment. Neutrophils are highly implicated in the pathogenesis of AAA, driving the local inflammatory reaction, including NET release, as well as promoting intraluminal thrombus formation, all mechanisms which are harmful for the integrity of the aortic wall^45,46^. Because CD73 expression is massively reduced in neutrophils under angiotensin II treatment, these cells in wildtype animals also exhibit a type of CD73^−/−^ phenotype, that is certainly associated with a proinflammatory milieu, which may explain why the CD73^−/−^ animals do not show increased inflammation compared with the wildtype strain. On the other hand, a randomized, double‐blind, placebo‐controlled study from Hakovirta et al. in 2022 reported that up-regulation of CD73 was significantly associated with improved survival after an emergency aortic reconstruction upon AAA rupture, suggesting CD73 as a potential target for a more beneficial outcome post-surgery^47^.

Formation of aortic disease is marked by different manipulable and non-manipulable risk factors. The two major non-manipulable risk factors are gender and age. Men are more often affected by this disease than women, especially the elderly population^48^. However, both factors for disease development were not influenced by the lack of CD73. Furthermore, we could not detect any CD73-related age or gender specific changes in vessel physiology or immune cell distribution. Thus, our results might be of particular interest because it indicates that inhibition of CD73 will not interfere with these risk factors. Furthermore, they might be also of relevance for several clinical trials using the inhibition of CD73 for cancer treatment^49–51^. In these cases, patients subjected to CD73 inhibition should not be at an enhanced risk to develop aortic disease.

## Methods

### Animals

#### General

Animal experiments were approved by the institutional review board “Landesamt für Natur, Umwelt und Verbraucherschutz Nordrhein-Westfalen” and were performed in accordance with the ARRIVE guidelines as well as the national guidelines on animal care (Az: 81-02.04.2018.A468 and Az: 81-02.04.2021.A401). Male and female mice (WT and CD73^−/−^; 3 and 6 months of age) used in this study were bred and housed in the central animal facility of the Heinrich Heine University (Düsseldorf, Germany) and fed with a standard chow diet and received tap water ad libitum.

#### Implantation of osmotic minipumps

The pump body was filled up with angiotensin II (6 µg/µl solved in NaCl) and placed in an Eppendorf Safe-lock tube (Eppendorf Quality, Hamburg, Germany), which was filled with 0.9% NaCl and placed overnight in a 37 °C warming cabinet. 30 min before operation, mice received i.p. 0.1 mg/kg buprenorphine. The pump was inserted subcutaneously in a minimally invasive procedure, where the mouse was anesthetized by 3% isoflurane using a homemade nose mask. The back of the head was shaved off, followed by the preparation of a subcutaneous pocket using blunt scissors, inserting the pump and then suturing the skin (6-0 Suture) with a crisscross suture. This process took about 5–7 min per mouse. The pump was then left in place for a period of 10 days releasing an average amount of 1.5 µg angiotensin II per hour.

#### Isolation of the aorta

The mice were euthanized by injection of 100 mg/kg of ketamine and 10 mg/kg of xylazine. Subsequently, the abdomen and thorax were opened and the left common iliac artery was cut through followed by a puncturing of the left ventricle with 20 ml of a PBS solution (Carl Roth, Karlsruhe, Deutschland). Approximately 10 min before, the mice were injected intraperitoneally with a 200 µl 0.2 molar heparin-PBS solution. All organs were removed and the aorta dissected. The surrounding perivascular fat was carefully removed from the aorta and excised almost 1 mm below the aortic arch and 1 mm above the bifurcation of the aorta.

#### Immune cell isolation from the aorta

The immune cells from the freshly excised aorta were isolated via a gentle MACS dissociation using the tumor dissociation kit and the manufacturer’s protocol. In short, enzyme mix was mixed with RPMI in a C-tube and the aorta was digested over 41 min. Afterwards, debris was removed via a 40 µm cell filter and the cells were washed with MACS buffer.

### Flow cytometry

#### General

Flow cytometry was performed at a FACS Canto II (BD Biosciences, USA). Cells were gated with appropriate forward and side scatter settings and thresholds for excluding debris. To omit dead cells, samples were stained with 1 µg/ml DAPI (4′,6-Diamidin-2-phenylindol, Merck). For analysis, cells were gated with FACS Diva software and the mean fluorescence intensities and/or the number of positive cells were determined, depending on the experiment.

#### Murine immune cells

The individual mouse immune cell populations were discriminated by antibody staining. FcR-blocking solution was applied for 10 min at 4 °C. After washing with 200 µl MACS buffer, staining against CD45 (BD Biosciences, clone 30-F11) and CD11b (Biolegend, clone M1/70) for total immune cells (CD45^+^), myeloid cells (CD45^+^ + CD11b^+^) and lymphoid cells (CD45^+^ + CD11b^−^) was performed. Furthermore, ectonucleotidases CD38 (BD Bioscience, clone 90/CD38), CD39 (BD Bioscience, clone Y23-1185) and CD73 (Biolegend, clone TY/11.8) were stained on the three main immune cell populations. Cells were stained for 20 min at 4 °C, followed by washing with 200 µl MACS buffer.

#### Data analysis

Flow cytometry data sets were analyzed via FlowJo_10.8.0.

### Histology

#### Haematoxylin/eosin (H&E) staining of the aorta

The aorta was fixed with 10% formaldehyde, embedded in paraffin, and cut into 5 µm sections. These sections were fixed at 70 °C for 1 h. Subsequently, the sections were deparaffinized, washed in PBS, and hydrated in distilled water for 1 min. Hematoxylin staining was performed for 1 min, followed by differentiation in 1% hydrochloric acid alcohol solution. The staining was then blued in running tap water for 10 min. Eosin staining was carried out for 1 min prior to the dehydration process using ethanol in increasing concentrations (70%, 96%, absolute ethanol) and Roticlear. Finally, the sections were mounted with Rotimount and investigated under the microscope. The thickness of the aortic walls was determined in slices of the aorta from comparable levels at four locations using ImageJ and subsequently averaged.

### Magnetic resonance imaging (MRI)

#### General

All experiments were performed using a vertical 9.4 T Bruker AVANCE^III^ Wide Bore NMR spectrometer (Bruker, Rheinstetten, Germany) operating at frequencies of 400.21 MHz for ^1^H measurements using a Bruker microimaging unit Micro 2.5 with actively shielded gradient sets (1.5 T/m). Data was acquired using a 25 mm ^1^H resonator. Mice were anaesthetized with 1.5% isoflurane, kept at 37 °C and placed within the resonator. Respiration was monitored by means of a pneumatic pillow positioned at the animal’s back and vital function was acquired by a M1025 system (SA Instruments, Stony Brook, NY, USA).

#### Abdominal aorta

After axial pilot scans the complete aorta was covered in longitudinal direction over 36 sections with 12 sections below the lower kidney vessel and 24 sections above. Scan details for the angio FLASH scan: ^1^H fast low angle shot (FLASH); repetition time (TR) = 10,000 ms, FOV = 2.56 × 2.56 cm^2^, matrix: 192 × 192, slice thickness (ST) 0.5 mm, acquisition time (TAcq) 5 min.

#### Data analysis

MR data were analyzed using in-house developed software modules based on the LabVIEW package (National Instruments, Austin, TX) as described previously^52,53^.

### Ultrasound

#### General

The mouse was anesthetized with 3% isoflurane in a chamber and placed on a thermostatically controlled heating pad in supine position with the paws taped over the ECG electrodes attached to the table. Anesthesia was maintained with 1.8% isoflurane in 100% oxygen with a flow of 0.8 l/min administered by means of a face mask connected to a coaxial circuit. Respiratory gating was derived from ECG. A small-diameter temperature probe was inserted through the anus and a depilatory cream applied on the abdominal region by using cotton tipped applicators in a circular motion; the cream remained on the skin for 30 s before being removed by using a gauze soaked in water. A colorless aqueous warmed ultrasonic gel (Supragel^®^, LCH, France) without any air bubbles was applied between the skin and the transducer (MX 550) (Vevo 3100 FUJIFILM, VisualSonics). The heating pad was tilted backwards (approx. 30°) and the transducer was placed on the abdomen. Using the transducer (40 MHz), the aorta was located transversally in an epigastric angle. The transducer was rotated 90° clockwise, to locate the aorta sagittally. The point between the celiac artery and the cranial mesenteric artery was the standard measuring point for all mice. Finally, the ultrasonic gel and the rectal probe were removed from the mouse and the animal was awakened from anesthesia under a heat lamp and observation.

#### Strain

The aortic strain, a measurement of vascular elasticity, which would reflect the stiffness of the aorta, can be calculated using a certain formula (½ × [(Diameter_systole_/Diameter_diastole_)^2^ − 1] × 100%), from the parameters measured in the M mode, where the layers of the aorta are displayed and the systolic and diastolic diameters could be measured. It is crucial that the ultrasound waves intersect the aorta perpendicularly.

#### Blood flow velocity

The peak systolic, end diastolic and mean velocity can be deduced using the pulse wave mode. A measurement window appears on the screen and was adjusted in an angle of at least 60° to the vascular course. The section between the celiac trunk and the superior mesenteric artery was fitted inside that window and was adjusted so that one half to two-thirds of the vessel diameter was analyzed. The pulse repetition frequency and gain were adjusted until the velocity of the blood flow was best measured and could be saved.

#### Data analysis

The analysis of the imaging data was done with the help of the software Vevo LAB.

### Continuous blood pressure measurements by radio telemetry

Blood pressures were measured by radio telemetry (PA-C10, Data Sciences International, Saint Paul, MN, USA). Pressure-sensing catheters were implanted in the left carotid artery under intraperitoneal anaesthesia (ketamine (100 mg/kg) and xylazine (10 mg/kg) as described previously^54^. In brief, the left common carotid artery was dissected free, mobilized, and cannulated. The catheter was then advanced to the point where the small notch at the end of the tube was at the vessel opening. The catheter was then fixed and the transmitter was placed subcutaneously. After seven days of recovery and reestablishment of diurnal blood pressure variations, measurements were collected continuously with sampling every 20 min for 10-s intervals throughout the experimental period.

### Ultra-performance liquid chromatography—degradation of ATP

Isolated murine immune cells from CD73 WT and CD73^−/−^ mice were washed once with HBSS buffer. Afterwards, cells were resuspended in 100 µl HBSS with 20 µM ATP. After 30 min of incubation at 37 °C, cells were centrifuged at 300*g* for 5 min and 50 µl of the cell-free supernatant was transferred into UPLC vials. The degradation of ATP and the upcoming products (ADP, AMP) were applied to an ACQUITY UPLC Bio H-Class System equipped with a Cortecs C18 + UPLC column (30 × 150 mm, particle size 1·6 µm) (Waters, Milford, MA, US). Purine separation was performed as previously described^55^ using a linear gradient of buffer A (150 mM KH_2_PO_4_/150 mM KCl, pH 6) and buffer B (150 mM KH_2_PO_4_/150 mM KCl/15% acetonitrile, pH 6). Absorbance was measured at 254 nm.

### Statistical approach

Due to high variances of the model utilized (see Supplemental Table 1), we decided to use a pooling statistical approach, where all animals were used for all quantifications, independent of treatment (basal/angiotensin II), genetic background (wildtype/CD73^−/−^), age (3 months/6 months) or sex (male/female) to analyze the main effects independently of the individual subgroups, which allowed us to keep mouse numbers low in line with the three Rs principle. To this end, mice were divided into groups according to the main variables: CD73 background, age, sex, and angiotensin II treatment. These main variables were first tested for normal distribution independently of any interactions with each other, then in different interactions, both using the Shapiro–Wilk test. Non-normally distributed data was tested for significance by the Mann–Whitney *U* test or the Kruskal–Wallis test. For normally distributed data we used the Student’s *t* test and ANOVA. P values less than 0.05 were considered significant.

### Supplementary Information

Supplementary Information.

## Data Availability

All data generated or analyzed during this study are included in this published article (and its Supplementary Information files).
