# Supplementary material for: CD73 deficiency does not aggravate angiotensin II-induced aortic inflammation in mice
Source: Sci Rep. 2023 Oct 10;13:17125. doi: 10.1038/s41598-023-44361-7 (PMC10564884; doi:10.1038/s41598-023-44361-7)
Supplement: Supplementary file 1 — Supplementary Information. [file 41598_2023_44361_MOESM1_ESM.pdf]

	Immune cells	Myeloid cells	Lymphoid cells	Macrophages	Neutrophils	T cells	B cells	Strain	Velocity
CD73 wildtype basal male 3 months	1492±791	503±357	931±600	15±15	489±347	285±145	84±39	0.23±0.03	344±39
CD73 wildtype d10 male 3 months	2949±892	2181±854	726±297	299±126	1896±739	269±114	82±20	0.15±0.1	257±56
$CD73^{-/-}$ basal male 3 months	1728±1435	1015±1125	537±388	145±103	835±948	190±149	49±24	0.15±0.08	356±82
$CD73^{-/-}$ d10 male 3 months	3172±850	2204±809	892±242	423±237	1556±610	197±79	119±54	0.1±0.1	247±68
CD73 wildtype basal female 3 months	1698±794	1035±467	335±104	79±41	1085±740	269±131	62±30	0.17±0.14	290±34
CD73 wildtype d10 female 3 months	3361±2284	1923±1261	1060±1134	33±39	1708±990	652±641	175±215	0.21±0.06	202±89
$CD73^{-/-}$ basal female 3 months	1276±717	811±562	322±168	48±20	874±679	432±677	198±357	0.15±0.08	239±54
$CD73^{-/-}$ d10 female 3 months	3039±1578	2004±988	745±658	52±27	1291±863	304±330	80±64	0.12±0.05	146±38
CD73 wildtype basal male 6 months	657±400	247±188	368±293	25±36	221±193	256±241	43±32	0.12±0.06	182±98
CD73 wildtype d10 male 6 months	4238±2005	1976±1524	2117±1224	27±44	1944±1473	606±330	345±178	0.18±0.07	327±80
$CD73^{-/-}$ basal male 6 months	950±574	423±213	490±342	8±5	412±206	248±171	106±77	0.11±0.08	192±72
$CD73^{-/-}$ d10 male 6 months	2944±1025	1326±408	1486±664	51±47	1265±375	600±321	366±325	0.27±0.1	328±72
CD73 wildtype basal female 6 months	845±302	558±291	223±50	33±11	526±293	131±25	66±29	0.19±0.03	303±103
CD73 wildtype d10 female 6 months	1800±1004	1268±681	403±288	40±3	1221±681	289±246	95±43	0.17±0.05	166±60
$CD73^{-/-}$ basal female 6 months	1950±451	1486±357	341±68	276±64	1167±285	199±44	91±22	0.12±0.06	267±45
$CD73^{-/-}$ d10 female 6 months	5291±4799	1951±1612	2836±3654	14±4	972±190	464±104	423±198	0.11±0.05	122±15

Supplemental Table 1

Shown is the mean ± SD of the different treated groups. Depicted are immune cell numbers as well as strain and blood flow velocity.

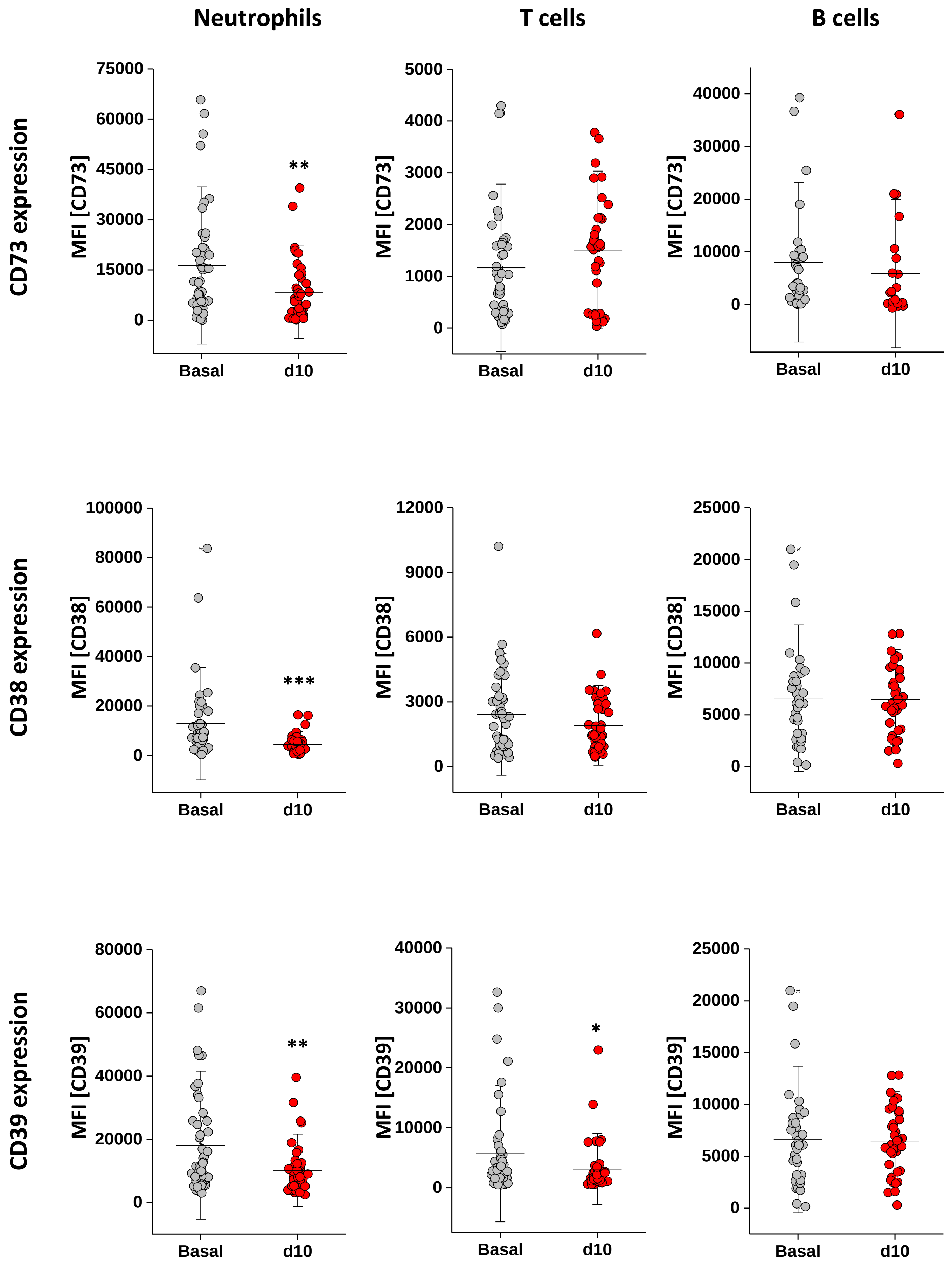

Supplemental Figure S1

The cell surface expression of CD73, CD39 and CD38 were analyzed for neutrophils, B- and T-cells isolated from the aorta under basal conditions or 10 days after AngII treatment. While neutrophils are marked by a decrease of all three ectoenzymes after AngII treatment, T- and B-cells show no differences. Only CD39 is downregulated for T cells after AngII treatment. All data sets are mean values +- SD of n = 41 - 51. * = $p < 0.05$, ** = $p < 0.01$ and *** < 0.001 verified by Mann-Whitney U test.

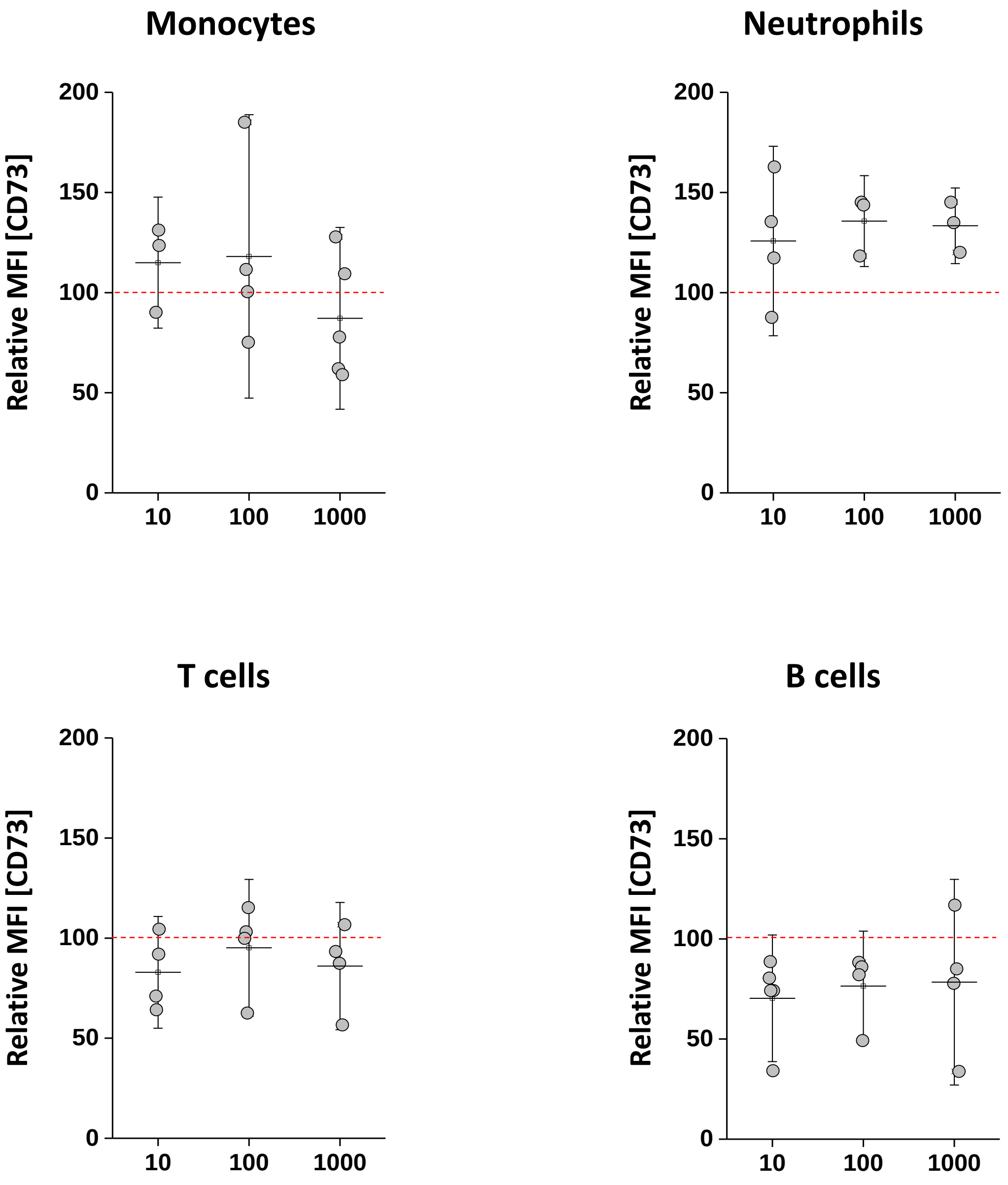

Supplemental Figure S2

Isolated immune cells from the blood were incubated ex vivo for 1 hour with different concentrations of AngII followed by determination of the CD73 expression via flow cytometry to investigate any influence of AngII on CD73 surface expression. Normalization of the AngII treated groups to the non-treated ctrl group revealed no significant increase in the surface expression of CD73. All data sets are mean values ± SD of n = 3 - 5.

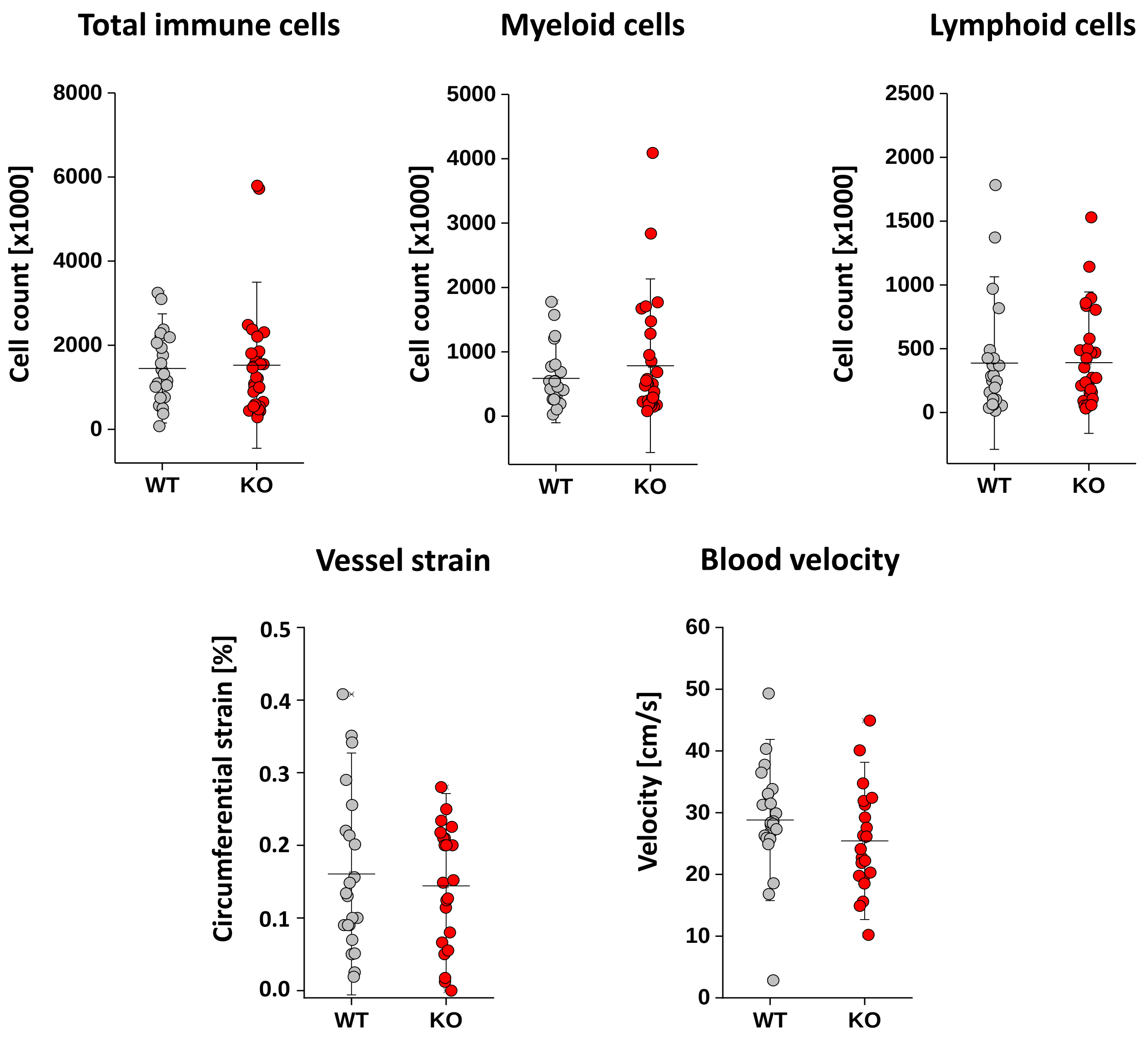

Supplemental Figure S3

Comparison of aortic immune cells, vessel strain as well as blood flow velocity in CD73 WT and KO mice at baseline prior infusion of Angiotensin II. All data sets are mean values ± SD of n = 21 - 30.

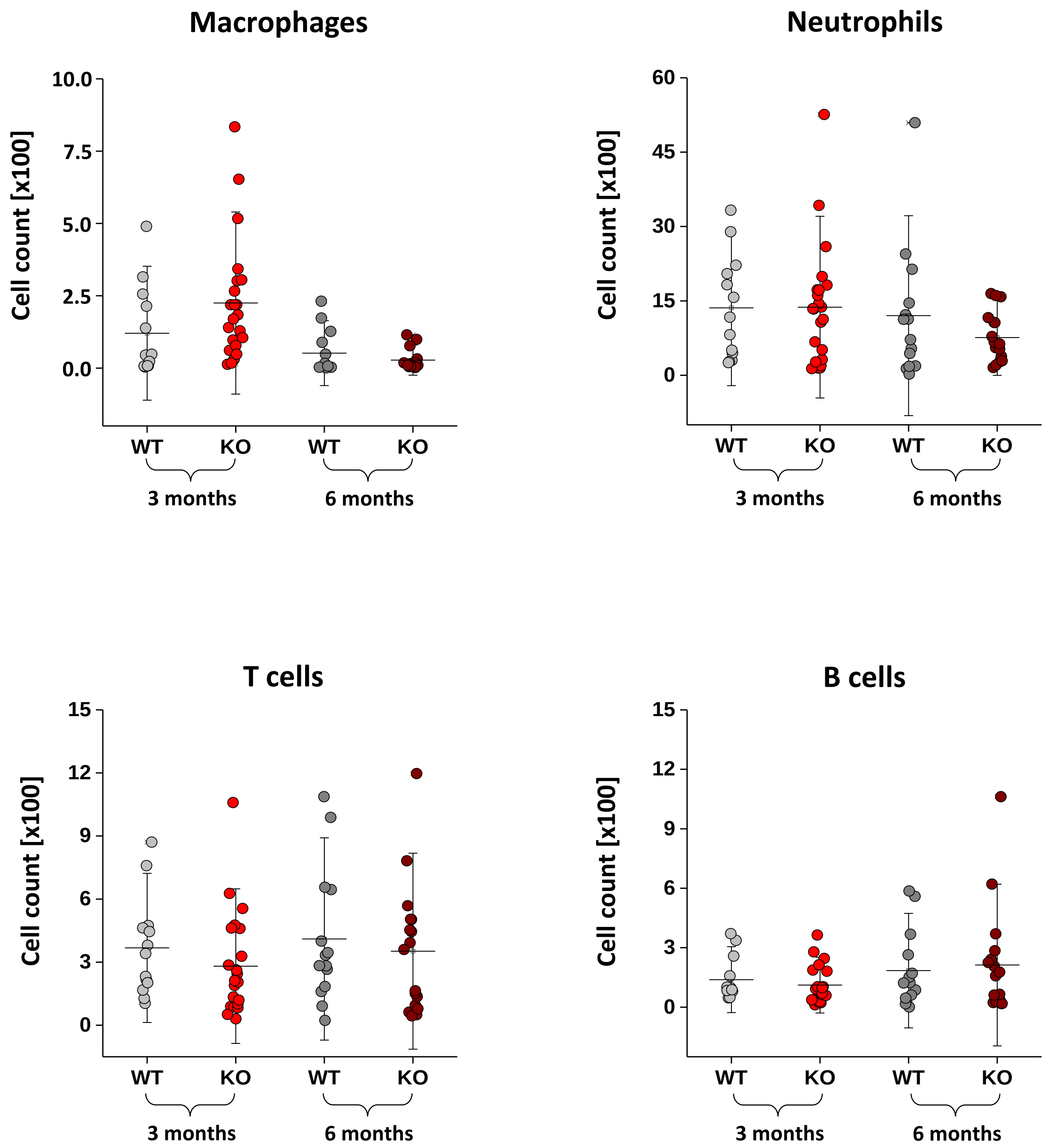

Supplemental Figure S4

Flow cytometric analysis of aortic macrophages, neutrophils, T and B cells in CD73 WT and KO mice with an age of 3 and 6 months. We could not detect any significant change between the groups analyzed. All data sets are mean values ± SD of n = 13 - 22.

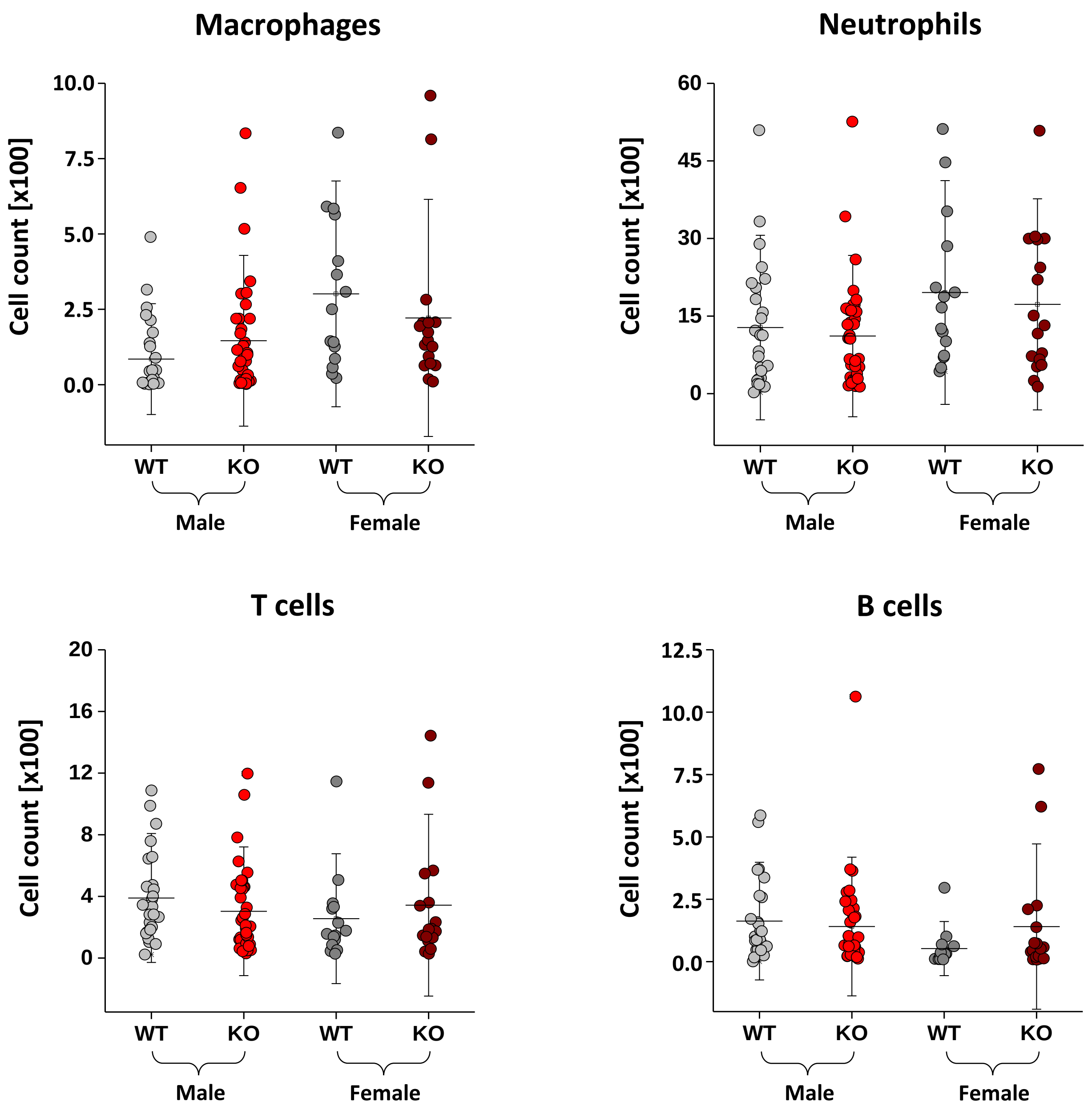

Supplemental Figure S5:

Total number of macrophages, neutrophils, T and B cells were determined within aortas from male and female CD73 WT and KO mices. We could not detect any significant change between the groups analyzed. All data sets are mean values ± SD of n = 13 - 22.

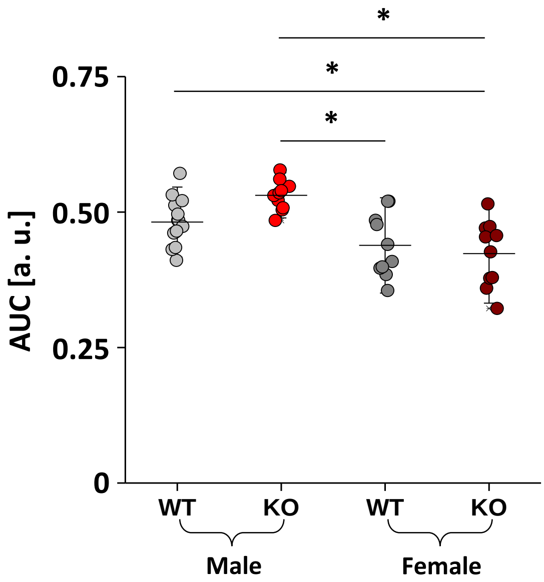

Supplemental Figure S6:

Analysis of the area under the curve (AUC) of the vessel lumen (see fig. 5D) of male and female CD73 WT and KO mice. Female CD73$^{-/-}$ mice are characterized by a significant drop in the AUC compared to male mice, while female CD73 wildtype mice show only a significant reduction in AUC compared to male CD73$^{-/-}$ mice. All data sets are mean values ± SD of n = 10 - 14. * = $p < 0.05$ verified by one-way ANOVA.
